# When all nails are involved: An atypical nail emergency

**DOI:** 10.1016/j.jdcr.2026.09.017

**Published:** 2026-09-17

**Authors:** Kenza Khachani, Kaoutar Znati, Mariame Meziane, Nadia Ismaili, Laila Benzekri

**Affiliations:** a Department of Dermatology, Mohammed V University in Rabat, Ibn Sina University Hospital, Rabat, Morocco; b Department of Pathology, Mohammed V University in Rabat, Ibn Sina University Hospital, Rabat, Morocco

**Keywords:** anonychia, lichen planus, nail dystrophy, pterygium, scarring

## Clinical case

A 41-year-old woman with a history of type 2 diabetes and first-degree parental consanguinity, presented with a 10-month history of rapidly progressive nail dystrophy affecting both hands and feet, significantly impairing her quality of life.

Examination revealed diffuse and severe nail involvement, with anonychia, erosions, and nail bed hypertrophy of all toenails. On the hands, findings included longitudinal ridging and striations (onychorrhexis), anonychia with erosion of the right fifth fingernail, and dorsal pterygium of the left thumb and fifth fingernail (Fig 1). Subungual hyperkeratosis and melanonychia affected the right thumb and the right fourth fingernail. Onychoscopy showed longitudinal fissures and ridges, without tortuous capillaries in the proximal nail fold (Fig 2).

**Fig 1:**
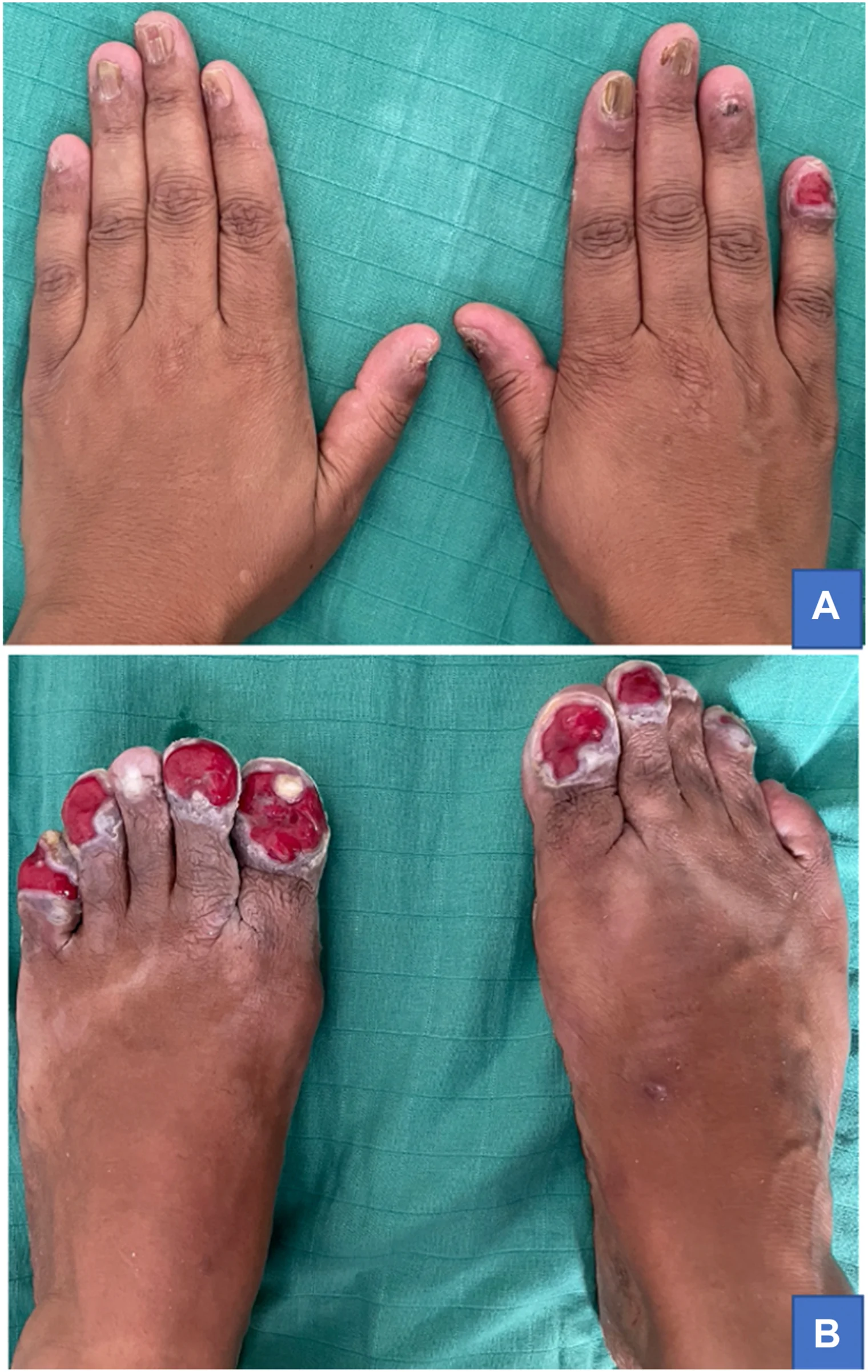
Clinical presentation of diffuse and severe lichen planus of fingernails **(A)** and toenails **(B)**.

**Fig 2:**
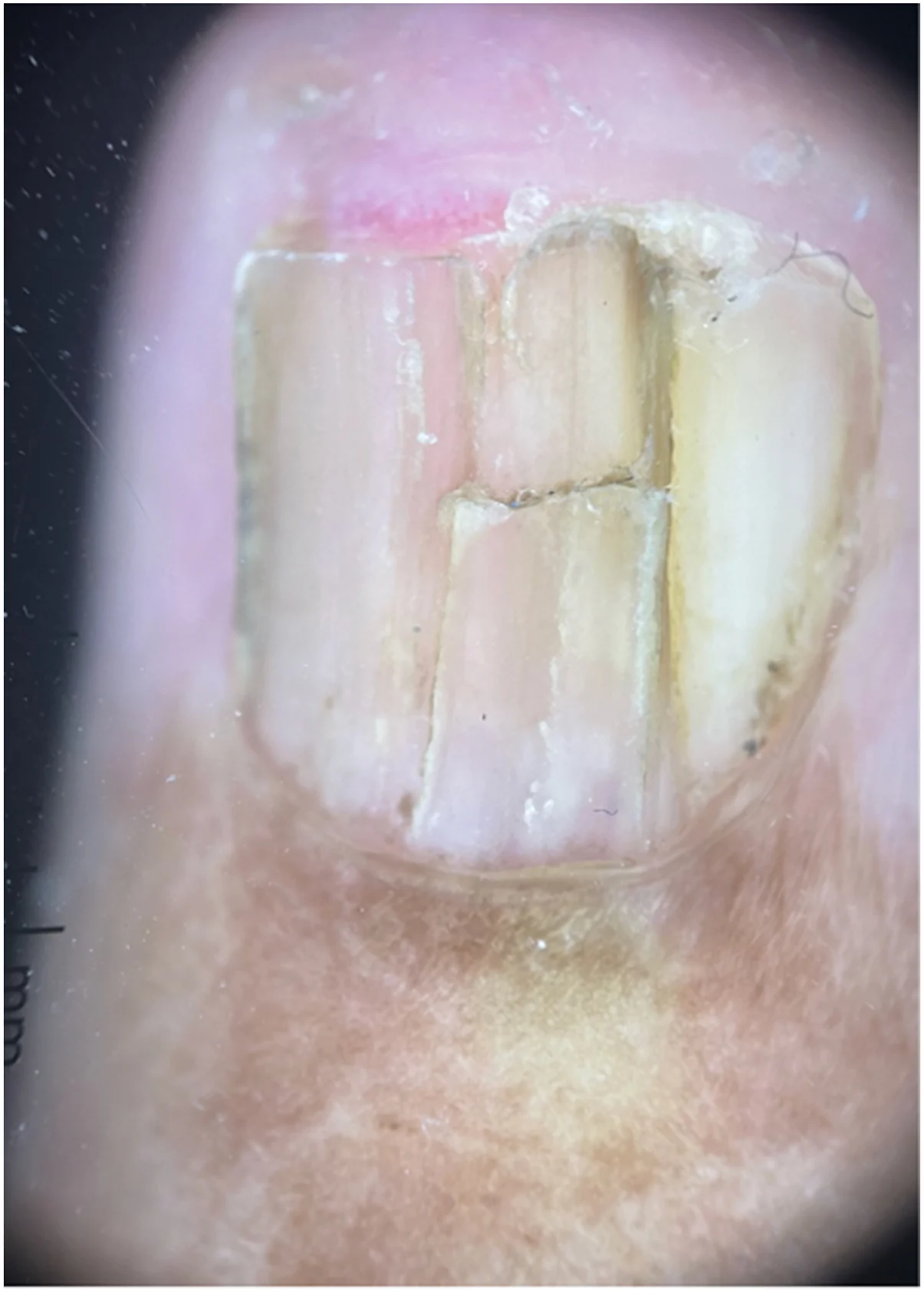
Dermoscopy of nail Lichen planus: longitudinal fissures and ridges, without tortuous capillaries in the proximal nail fold.

Oral examination showed erosions with leukoplakia and gray papules on the tongue dorsum (Fig 3). No cutaneous, hair or genital lesions were noted.

**Fig 3:**
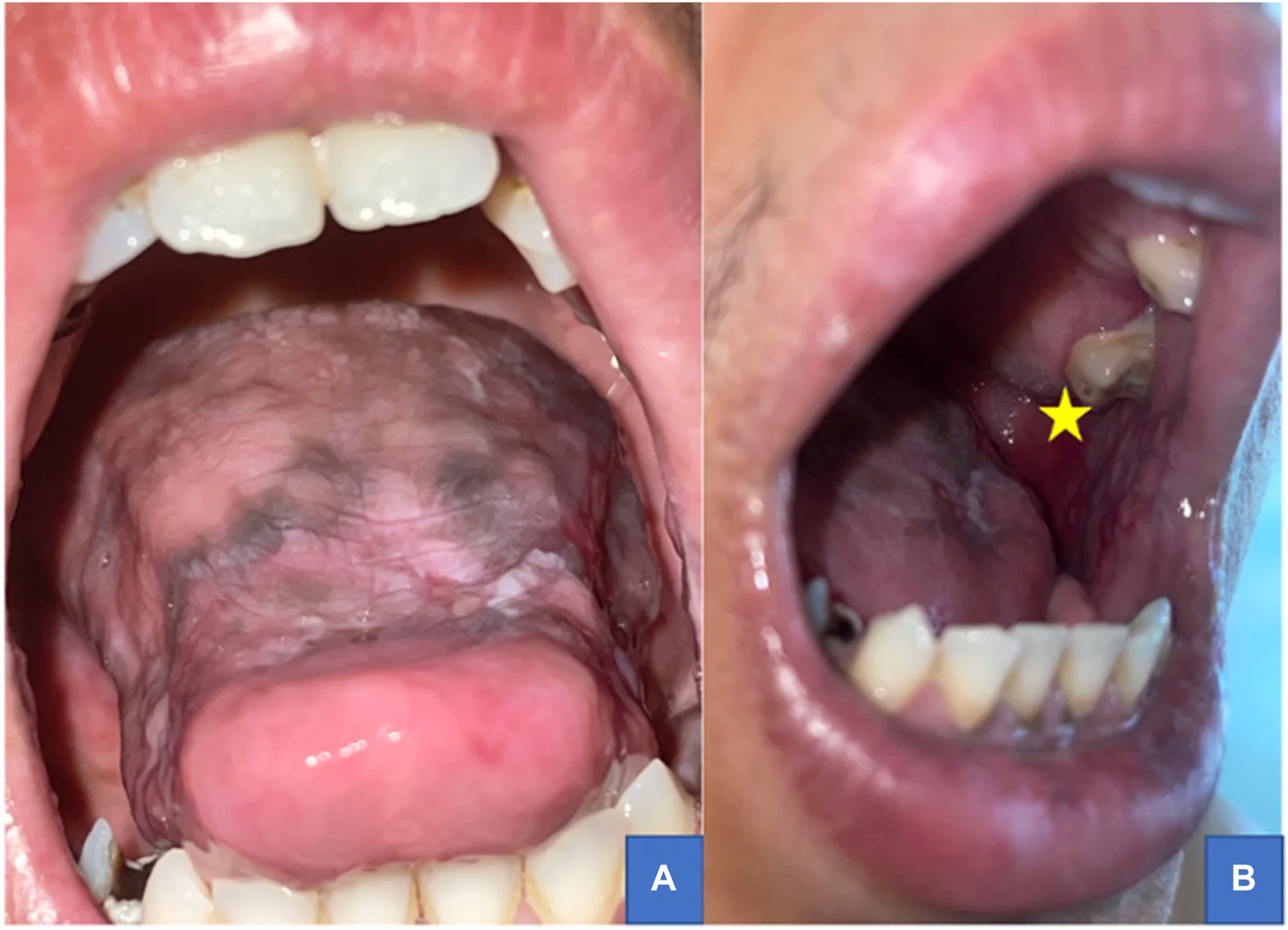
Oral involvement of Lichen planus: **A,** Leucoplakia and gray papules on the tongue dorsum. **B,** Mucosal erosion (*yellow star*).

Nail matrix and bed histology showed an acanthotic epidermis with a band-like subepidermal lymphocytic infiltrate, basal layer degeneration, melanophages and apoptotic bodies, consistent with a lichenoid pattern (Fig 4). Immunohistochemistry revealed a predominant CD8+ T-cell infiltrate with CD20+ B-lymphocytes. Direct immunofluorescence was negative.

**Fig 4:**
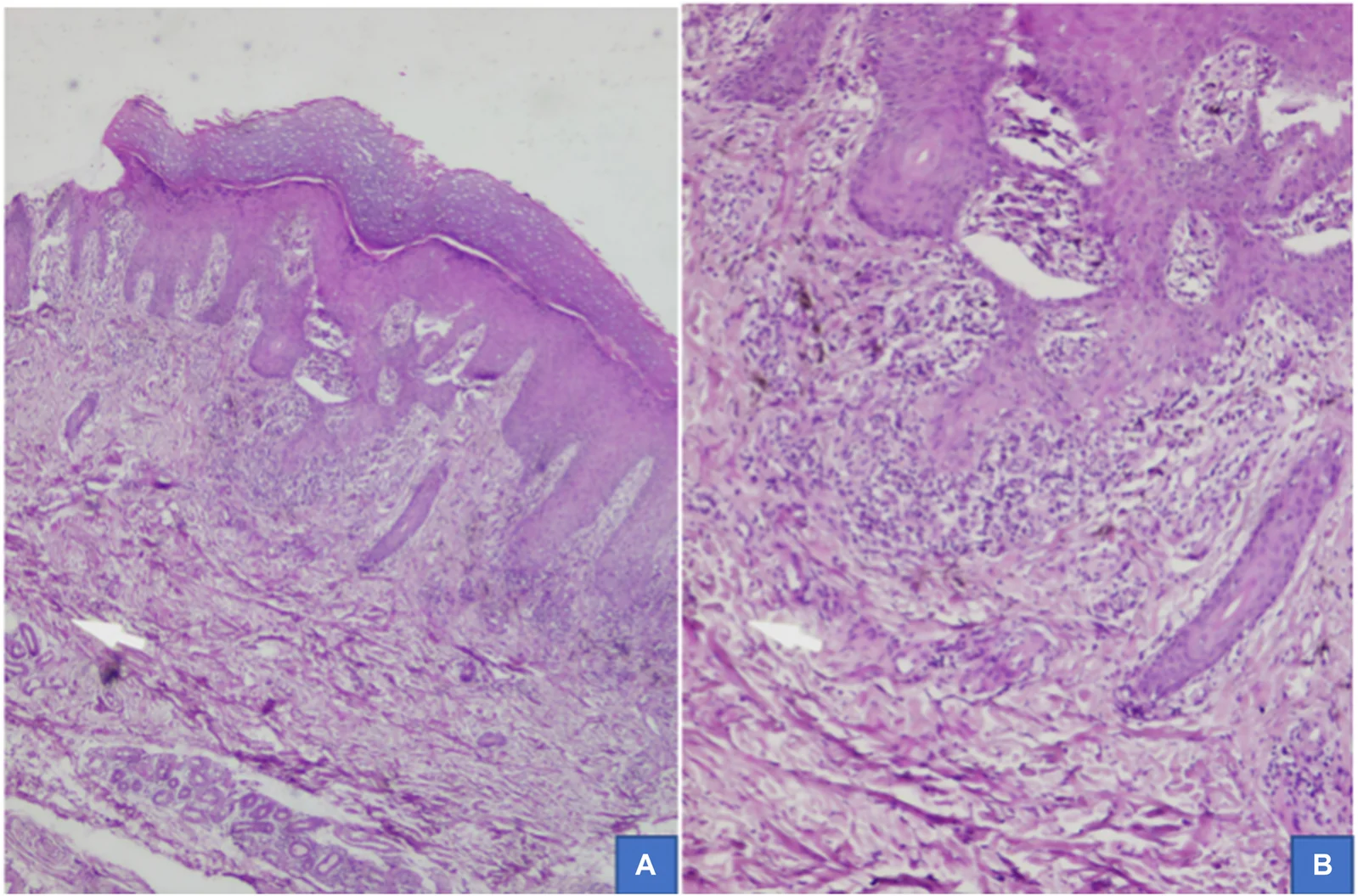
Histopathology of lichen planus (H&E stain). **A,** Low magnification showing an acanthotic epidermis with a band-like subepidermal lymphocytic infiltrate. **B,** Higher magnification showing basal layer degeneration, melanophages, and apoptotic bodies at the dermoepidermal junction.

The patient was treated with oral corticosteroids (0.5 mg/kg/d), light emitting diode phototherapy, and healing cream, with clinical improvement.

**Question: What is the most likely diagnosis?**

**A.** Dyskeratosis congenita
**B.** Lichen planus
**C.** Psoriasis
**D.** Onychomycosis with secondary onychomadesis
**E.** Pemphigus (vulgaris/paraneoplastic)

## Discussion

### Diagnosis: Lichen planus

Nail involvement occurs in approximately 10% of patients with lichen planus and constitutes a true nail emergency, as delayed management carries a substantial risk of irreversible scarring.^1^^,^^2^ Such a diffuse presentation is rarely reported. Nail lichen planus is most frequently associated with oral lesions (25% of cases), as observed in our patient.^3^

Matrix involvement leads to nail plate thinning, longitudinal ridging and fissuring, that may progress to dorsal pterygium or anonychia. Nail bed involvement manifests as onycholysis and subungual hyperkeratosis. Both were present in our patient.

Based on nail plate changes (fissuring, splitting length, onycholysis, lunula erythema, pterygium and anonychia), severity is classified as: mild, moderate or severe.^3^ Our patient’s presentation was consistent with severe disease, characterized by complete fissuring, pterygium and anonychia.

The diffuse, severe and rapidly progressing lesions raised several differential diagnoses. Dyskeratosis congenita was considered, but this condition classically presents in childhood, with nail dystrophy typically appearing before the age of 10 years, followed by oral leukoplakia and reticulated skin pigmentation. Onychomycosis with onychomadesis was excluded based on the clinical course and negative mycological sampling. Pemphigus, either vulgaris or paraneoplastic, was considered given the erosive mucosal and periungual involvement; however, the absence of bullous lesions, negative direct immunofluorescence, and underlying neoplasia argued against this diagnosis. The absence of nail pitting, salmon-patch discoloration, erythematosquamous plaques, or pustules argued against psoriasis.

Systemic corticosteroids may be considered for fewer than 3 nails when severe, and were justified here by the diffuse and severe nail involvement with concomitant erosive oral lesions, with a favorable outcome.^1^, ^2^, ^3^

This case highlights the destructive potential of nail lichen planus and underscores the importance of early diagnosis and prompt treatment to limit irreversible functional and psychosocial sequelae.

## Conflicts of interest

None disclosed.
